# Longitudinal associations of insulin resistance with change in bone mineral density in midlife women

**DOI:** 10.1172/jci.insight.162085

**Published:** 2022-10-24

**Authors:** Albert Shieh, Gail A. Greendale, Jane A. Cauley, Preethi Srikanthan, Arun S. Karlamangla

**Affiliations:** 1 Department of Medicine, David Geffen School of Medicine at University of California, Los Angeles, Los Angeles, California, USA.; 2 Department of Epidemiology, School of Public Health, University of Pittsburgh, Pittsburgh, Pennsylvania, USA.

**Keywords:** Bone Biology, Bone disease, Insulin

## Abstract

**BACKGROUND:**

The effects of insulin resistance on bone mineral density (BMD) are unclear.

**METHODS:**

In Study of Women’s Health Across the Nation (SWAN) participants, we used multivariable regression to test average insulin resistance (homeostatic model assessment of insulin resistance, HOMA-IR) and rate of change in insulin resistance as predictors of rate of change in lumbar spine (LS) and femoral neck (FN) BMD in 3 stages: premenopause (*n* = 861), menopause transition (MT) (*n* = 571), and postmenopause (*n* = 693). Models controlled for age, average BW, change in BW, cigarette use, race and ethnicity, and study site.

**RESULTS:**

The relation between HOMA-IR and BMD decline was biphasic. When average log_2_HOMA-IR was less than 1.5, greater HOMA-IR was associated with slower BMD decline; i.e., each doubling of average HOMA-IR in premenopause was associated with a 0.0032 (*P* = 0.01, LS) and 0.0041 (*P* = 0.004, FN) g/cm^2^ per year slower BMD loss. When greater than or equal to 1.5, average log_2_HOMA-IR was not associated with BMD change. In women in whom HOMA-IR decreased in premenopause, the association between the HOMA-IR change rate and BMD change rate was positive; i.e, slower HOMA-IR decline was associated with slower BMD loss. In women in whom insulin resistance increased in premenopause, the association was negative; i.e, faster HOMA-IR rise was associated with faster BMD decline. Associations of average HOMA-IR and HOMA-IR change rate with BMD change rate were similar in postmenopause, but weaker during the MT.

**CONCLUSION:**

When it decreases, insulin resistance is associated with BMD preservation; when it increases, insulin resistance is associated with BMD loss.

**FUNDING:**

The SWAN has grant support from the NIH of the Department of Health and Human Services (DHHS) through the NIH National Institute on Aging (NIA), National Institute of Nursing Research (NINR), and Office of Research on Women’s Health (ORWH) (grants U01NR004061, U01AG012505, U01AG012535, U01AG012531, U01AG012539, U01AG012546, U01AG012553, U01AG012554, U01AG012495, and U19AG063720).

## Introduction

Although there is wide recognition that type 2 diabetes mellitus (DM2) is a risk factor for fractures (1), the effects of insulin resistance (a key pathophysiologic mechanism in DM2) on bone remain uncertain. In vitro, insulin signaling promotes osteoblast differentiation, proliferation, and function (2–4). However, in the in vivo models of insulin resistance, insulin signaling leads to expansion of bone marrow adipose tissue, decreased trabecular bone mineral density (BMD), and decreased cortical thickness (5). In states of insulin resistance, osteoblasts may also be resistant to insulin signaling (6, 7). Results from human studies of the relation between insulin resistance and BMD are similarly inconclusive, with studies reporting positive (8–11), negative (12–16), or no association (17–20). Notably, to our knowledge, all published human investigations on this topic are cross-sectional (8–20).

The objective of this study was, therefore, to examine the longitudinal associations of insulin resistance with BMD in midlife women before, during, and after the menopause transition (MT). BMD decreases rapidly in a 3-year window spanning 1 year before to 2 years after the final menstrual period (FMP). We define this period as the MT; premenopause (more than 1 year prior to the FMP) precedes the MT, and postmenopause (more than 2 years after the FMP) follows it (21). During premenopause, the average rate of BMD loss is 0, but BMD decreases in approximately 10% of women. During the MT, BMD decline accelerates and occurs at its greatest rate, which, on average, is 2.5% per year at the lumbar spine (LS) and 1.7% per year at the femoral neck (FN). In postmenopause, BMD continues to decline, but more slowly (mean, 1.1% per year at the LS and FN) (21). This study addressed 3 questions: Is the average level of insulin resistance during each midlife stage (premenopause, the MT, and postmenopause) associated with the rate of change in LS or FN BMD during that stage? Is the rate of change in insulin resistance during each midlife stage associated with the rate of change in LS or FN BMD during the same stage? Are stage-specific average levels of insulin resistance and change in insulin resistance independently related to change in LS or FN BMD?

This study was conducted in the Study of Women’s Health Across the Nation (SWAN), a United States–based, multicenter, longitudinal study of the MT with up to 15 repeated assessments of insulin resistance (approximated by homeostatic model assessment of insulin resistance [HOMA-IR]) and up to 17 serial BMD measures.

## Results

### Sample characteristics.

Table 1 presents the participant characteristics for the 3 analysis samples, each corresponding to 1 of the 3 midlife stages: premenopause (*n* = 861), the MT (*n* = 571), and postmenopause (*n* = 693). To be included in a stage-specific sample, participants needed at least 2 observations in that stage; an individual woman could be represented in 1–3 samples. In all 3 samples, approximately one-quarter of women were Black, slightly more than one-quarter were Chinese or Japanese, and slightly less than half were White. Mean age at the first premenopausal, MT, and postmenopausal visits were 45.44, 50.71, and 55.11 years, respectively. Average insulin resistance was greater in successive midlife stages: the geometric means of HOMA-IR at the first visit in each stage were 1.81 (premenopause), 2.03 (MT), and 2.19 (postmenopause), corresponding to the average log_2_HOMA-IR values of 0.86, 1.02, and 1.13, respectively. The geometric means of HOMA-IR across all visits in a midlife stage were 1.90 (premenopause), 2.15 (MT), and 2.31 (postmenopause), corresponding to log_2_HOMA-IR averages of 0.931, 1.111, and 1.210, respectively. Insulin resistance increased faster during the MT than in the other 2 stages. During the MT, the average rate of change in log_2_HOMA-IR was +0.117 per year (8.7% gain per year in HOMA-IR), which was twice as fast as in postmenopause (0.064 per year increase in log_2_HOMA-IR, or 4.2% growth per year in HOMA-IR). The rise in insulin resistance was slowest in premenopause (+0.024 per year in log_2_HOMA-IR, or 1.4% increase per year in HOMA-IR).

As expected, the rate of BMD change was most negative (meaning the fastest decline) during the MT than in the other 2 midlife stages: at the LS, average rates of BMD change were –0.0008, –0.0232, and –0.0056 g/cm^2^ per year during premenopause, the MT, and postmenopause, respectively. Corresponding rates of change in FN BMD were –0.0016, –0.0138, and –0.0062 g/cm^2^ per year. The mean length of stage-specific follow up (time from the first to last BMD measurements) for the 3 midlife stages were 4.411 years for premenopause, 1.627 years for the MT, and 6.849 years for postmenopause.

### Average insulin resistance level as predictor of annualized change in BMD.

**Figure 1:**
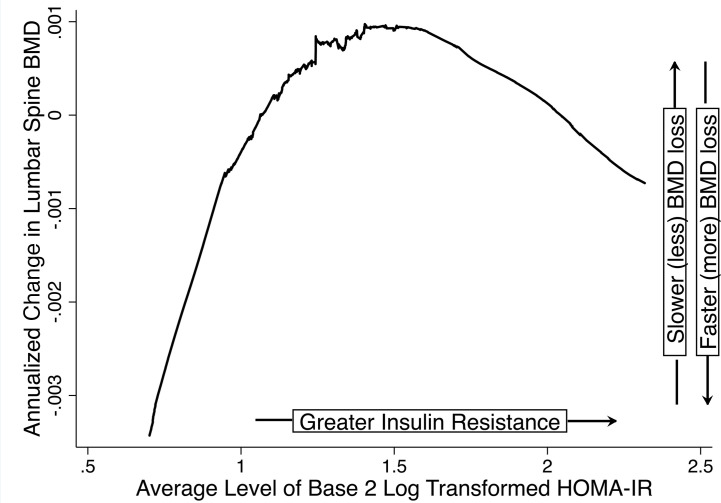
LOESS of annualized change in BMD versus average level of insulin resistance. For each midlife stage (premenopause, the MT, and postmenopause), we used LOESS to visualize the relation between annualized change in LS or FN BMD (g/cm^2^ per year) versus the average log_2_HOMA-IR. Annualized change in BMD was calculated as the difference between the last and first BMD values in a midlife stage divided by the number of intervening years. Thus, a more positive rate of change indicates slower BMD loss, and a more negative rate indicates faster BMD loss. Here, we show the LOESS plot for rate of change in LS BMD versus the average log_2_HOMA-IR level in premenopause. LOESS plots for the FN and the other midlife stages (menopause transition, postmenopause) were similar. Note the biphasic relation: when log_2_HOMA-IR was less than 1.5 (raw HOMA-IR = 2.82), greater log_2_HOMA-IR correlated with a more positive rate of change in BMD (slower bone loss); when log_2_HOMA-IR was greater than or equal to 1.5, greater log_2_HOMA-IR correlated with a more negative rate of change in BMD (faster bone loss).

Visual inspection of LOESS plots between average log_2_HOMA-IR and the rate of BMD change revealed that the relationship between average log_2_HOMA-IR and annualized change in LS or FN BMD was piecewise-linear in each of the 3 midlife stages, with a change in slope (knot) at an average log_2_HOMA-IR value of 1.5 (raw HOMA-IR = 2.82) (Figure 1). A total of 170 (19.7%, premenopause), 154 (26.9%, MT), and 204 (29.4%, postmenopause) women had average log_2_HOMA-IR equal to or greater than 1.5.

In stage-specific, multivariable linear regression of annualized rate of change in LS and FN BMD as a function of average log_2_HOMA-IR (operationalized using a 2-piece linear spline with knot at 1.5), and adjusted for age (years), race and ethnicity (Black, Chinese, Japanese, or White), cigarette use (yes/no), average BW (kg), annualized change in BW (kg/year), and study site, greater average log_2_HOMA-IR was associated with a more positive rate of BMD change (slower BMD decline) when average log_2_HOMA-IR was less than 1.5 (HOMA-IR < 2.82) but was not related to a change in BMD when average log_2_HOMA-IR was greater than or equal to 1.5 (HOMA-IR ≥ 2.82) (Table 2). In adjusted models, when average log_2_HOMA-IR was less than 1.5, each doubling of HOMA-IR was related to 0.0032 (*P* = 0.01) and 0.0041 (*P* = 0.004) g/cm^2^ per year slower BMD loss at the LS and FN, respectively, during premenopause. During the MT, at the FN only, each doubling of HOMA-IR was associated with a 0.0055 (*P* = 0.04) g/cm^2^ per year slower BMD decline. In postmenopause, each doubling of HOMA-IR was related to 0.0042 (*P* = 0.004, LS) and 0.0023 (*P* = 0.04, FN) slower decrease in BMD.

### Annualized change in insulin resistance as predictor of annualized change in BMD.

**Figure 2:**
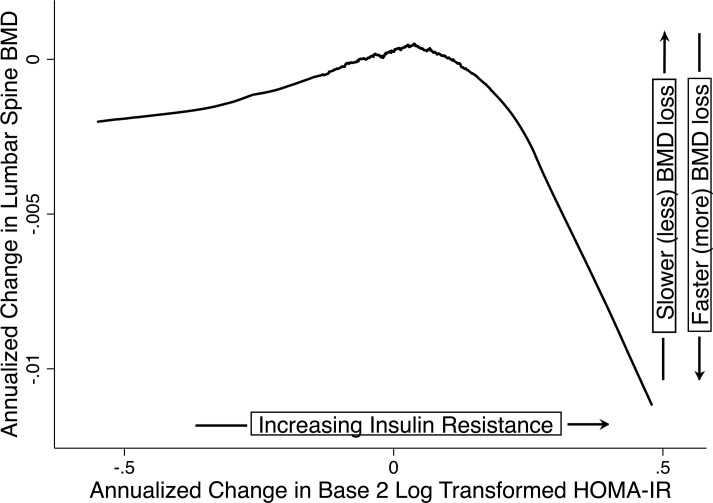
Annualized change in BMD versus annualized change in insulin resistance. For each midlife stage (premenopause, the MT, and postmenopause), we used LOESS to visualize the relation between annualized change in LS or FN BMD (g/cm^2^ per year) versus the annualized change in log_2_HOMA-IR. Annualized rates of change in BMD or log_2_HOMA-IR were calculated as the difference between the last and first BMD or log_2_HOMA-IR values in a midlife stage divided by the number of intervening years. A more positive rate of change indicates slower decrease in BMD or log_2_HOMA-IR, and a more negative rate indicates faster decline in BMD log_2_HOMA-IR. Here, we show the LOESS plot for change in LS BMD versus the change in log_2_HOMA-IR from premenopause. LOESS plots for the FN and the other midlife stages (menopause transition, postmenopause) were similar. Note the biphasic relation: when the change in log_2_HOMA-IR was less than 0 (insulin resistance decreasing), a more positive rate of change in log_2_HOMA-IR (slower decrease in insulin resistance) correlated with a more positive rate of change in BMD (slower bone loss); when the change in log_2_HOMA-IR was greater than or equal to 0 (insulin resistance increasing), a more positive rate of change in log_2_HOMA-IR (faster rise in insulin resistance) related to a more negative rate of change in BMD (faster bone loss).

In LOESS plots, the relationship between the annualized rate of change in log_2_HOMA-IR and the annualized rate of change in BMD was piecewise linear, with a knot at 0, implying that the relationship was different when the change in log_2_HOMA-IR was negative (insulin resistance decreasing) versus when the change in log_2_HOMA-IR was positive (insulin resistance increasing) (Figure 2). Insulin resistance increased in 520 (60.3%), 345 (60.1%), and 434 (62.6%) participants during premenopause, the MT, and postmenopause, respectively.

In stage-specific, multivariable linear regression of the annualized rate of change in LS and FN BMD as a function of the annualized rate of change in log_2_HOMA-IR (operationalized using a 2-piece linear spline with a knot at 0), when change in log_2_HOMA-IR was less than 0 (insulin resistance decreasing), a more positive rate of change in log_2_HOMA-IR greater than or equal to 0 (slower decline in insulin resistance) was associated with a more positive rate of change in BMD (slower bone loss); however, when the change in log_2_HOMA-IR was greater than or equal to 0 (insulin resistance increasing), a more positive rate of change in log_2_HOMA-IR was associated with a more negative rate of change in BMD (faster bone loss) (Table 3). During premenopause, when insulin resistance was decreasing, each SD slower decrease in log_2_HOMA-IR was related to a 0.0014 (*P* = 0.05, LS) and a 0.0030 (*P* = 0.005, FN) g/cm^2^ per year slower BMD loss. However, when insulin resistance was increasing, each SD faster rise in log_2_HOMA-IR was associated with a 0.0026 and 0.0034 g/cm^2^ per year faster decline in BMD at the LS (*P* = 0.001) and FN (*P* = 0.003), respectively. During the MT, a relation between change in insulin resistance and change in BMD was apparent only when insulin resistance was increasing and only at the LS: 0.0081 g/cm^2^ per year faster bone loss per SD faster gain in log_2_HOMA-IR (*P* = 0.005). In postmenopause, each SD slower decrease in log_2_HOMA-IR was associated with a 0.0029 (*P* < 0.001, LS) and 0.0019 (*P* < 0.001, FN) g/cm^2^ per year slower BMD loss, while each SD faster rise in log_2_HOMA-IR was related to a faster loss of LS BMD only (0.0021 g/cm^2^ per year, *P* = 0.03).

### Average insulin resistance level and annualized change in insulin resistance as predictors of annualized change in BMD.

The midlife stage-specific associations of average insulin resistance level and change in insulin resistance with the change in BMD change remained largely unchanged when both predictors were included in the same multivariable linear regression models, accounting for the same covariates as those in individual exposure models (Table 4).

## Discussion

To our knowledge, this is the first study to use data from a large, community-based cohort of diverse women to examine the longitudinal associations of insulin resistance with change in BMD during 3 midlife stages (premenopause, the MT, and postmenopause). In aggregate, we found that the average level of insulin resistance and rate of change in insulin resistance had nonlinear relations with the concurrent BMD change rate. At lower levels of insulin resistance, greater HOMA-IR was associated with slower BMD loss. For instance, each doubling of HOMA-IR was associated with 0.211 and 0.328 SD increments in the LS BMD change rate (less bone loss) in pre- and postmenopause, respectively. Similarly, when insulin resistance decreased over time, a slower decline in HOMA-IR was related to a slower decrease in BMD. For example, in pre- and postmenopause, each SD increment in the log_2_HOMA-IR change rate (smaller decline in insulin resistance) was related to 0.191 and 0.181 SD increments in the FN BMD change rate (less bone loss), respectively. In contrast, at higher levels of insulin resistance, HOMA-IR was not associated with BMD change. Correspondingly, when insulin resistance increased, a faster rise in HOMA-IR was related to faster BMD loss. Specifically, each SD increment in the log_2_HOMA-IR change rate (larger increase in insulin resistance) was associated with 0.183 and 0.164 SD decrement in the LS BMD change rate (more bone loss) in pre- and postmenopause, respectively.

A biphasic relation between insulin resistance and change in BMD is plausible; experimental data show that insulin has anabolic or catabolic actions on bone under different conditions and that bone itself can be an end-organ site of insulin resistance (2–7). At lower insulin concentrations, in vitro insulin signaling promotes osteoblast differentiation, proliferation, and function, supporting an anabolic effect (2–4). However, in insulin-resistant states, osteoblasts can become resistant to insulin signaling (6, 7); in vivo insulin signaling leads to expansion of bone marrow adipose tissue, decreased trabecular BMD, and decreased cortical thickness (5).

Unlike our longitudinal study, prior human investigations of the relation between insulin resistance and BMD have been cross-sectional. These studies have generated conflicting results with greater HOMA-IR or serum insulin being related to higher BMD levels (8–11), lower BMD levels (12–16), or having no association with BMD levels (17–20). One potential explanation for these discrepant findings is that, as we found in the current study, the relation between insulin resistance and BMD is nonlinear, but nearly all prior studies (8–11, 13–15, 17–19) tested for only linear associations. Thus, depending on the participants’ degree of insulin resistance, the relation between insulin resistance and BMD could vary from study to study. For example, studies showing that greater HOMA-IR or serum insulin correlated with higher BMD levels generally included fewer insulin-resistant (nondiabetic) participants (8–10). In contrast, greater insulin resistance related to lower BMD levels in most studies that consisted of individuals with greater degrees of insulin resistance (e.g., diabetics or post-transplant patients) (12–16). The Korean National Health and Nutrition Examination Survey tested for nonlinear relationships between serum insulin and BMD by level of insulin resistance; its findings, though cross-sectional, parallel those observed here. When HOMA-IR was in the lowest quartile, greater serum insulin was associated with higher BMD; in contrast, at higher HOMA-IR levels, greater serum insulin correlated with lower BMD (16). A second reason for the varied results from prior studies could be inconsistent handling of influential confounding variables, such as BMI (or BW); some (9–14, 16–20), but not all (8, 15, 22), analyses controlled for this covariate. Accounting for BMI is essential, as individuals with higher BMI are generally more insulin resistant and also have higher BMD. Indeed, greater HOMA-IR can be associated with higher BMD before controlling for BMI, be related to lower BMD (14), or be unrelated to BMD (10, 18–20) after adjustment for BMI.

We designed our analysis a priori to examine the longitudinal associations of HOMA-IR and rate of BMD change separately by midlife stage (premenopause, the MT, and postmenopause). This is because the trajectories of change in sex steroid hormones and BMD in each of these stages are different. During premenopause, estradiol (E2) levels and BMD are relatively stable (21, 23–25). E2 decreases rapidly during the MT, leading to rapid bone loss (21, 23–25). In postmenopause, E2 reaches its nadir and plateaus below premenopausal levels, accompanied by a slowing of BMD decline (21, 23–25). Because of these marked differences in endocrine and bone physiology between midlife stages, we postulated that the relations between HOMA-IR and BMD change could differ by stage. Indeed, our results suggest that insulin resistance has a smaller effect on bone during the MT than in pre- or postmenopause. We observed a positive relation between average HOMA-IR and BMD at the LS and FN (when insulin resistance is lower) in both pre- and postmenopause; however, during the MT, this association was apparent only at the FN. Similarly, the positive association of insulin resistance (when insulin resistance decreases) and the negative association of insulin resistance (when insulin resistance increases) with BMD were most uniform during pre- and postmenopause. In contrast, during the MT, decreasing insulin resistance was not associated with BMD change and increasing insulin resistance was related to more BMD loss at the LS only. We suggest that, because rapid E2 decline is a strong driver of BMD loss (26, 27), the influence of insulin resistance on bone is more difficult to discern during the MT. However, when E2 levels are relatively stable in pre- and postmenopause, and its effects on bone loss less dominant (28), the effect of insulin resistance on bone is more clearly detectable.

Our study has several limitations. First, very insulin-resistant individuals were not well represented because we excluded participants who were taking DM2 medications, which preclude HOMA-IR calculation, constraining generalizability. However, excluding those on diabetes medications removes potential confounding owing to the adverse effects of some diabetes medications on bone, suggesting that high levels of insulin resistance may indeed be detrimental to bone health. Second, due to the already complex design of this study, we did not explore the associations between insulin resistance level or change rate and measures of bone health other than BMD. DM2 is associated with lower bone turnover (29), worse trabecular microarchitecture (30), greater cortical porosity (31), and impaired bone material properties (32). Future studies will examine the longitudinal associations of insulin resistance with these important measures of bone health. Nonetheless, our results showing that a faster rise in insulin resistance relates to faster BMD loss suggest that, although those with type 2 diabetes often have higher BMD (33), continued increase in insulin resistance could mean more rapid bone loss and increased fracture risk.

To conclude, we report that the longitudinal associations of insulin resistance with BMD are nonlinear, and are more apparent in pre- and postmenopause than in the MT. Our findings suggest that insulin resistance may be beneficial for BMD preservation (slows BMD loss) when insulin resistance is low or decreases over time. In contrast, insulin resistance may be deleterious to BMD (hastens BMD loss) when insulin resistance increases over time. Further studies are needed to examine the associations of β cell function, insulin, and glucose with BMD loss and with markers of bone remodeling to elucidate the biological mechanisms underlying the relations observed in this study. Future analyses will also examine the longitudinal associations of insulin resistance with bone quality, bone strength, and the risk of fracture.

## Methods

SWAN is a multicenter, longitudinal study of 3,302 diverse, community-dwelling women. At study inception, participants were between 42–52 years and in premenopause (no change from usual menstrual bleeding pattern) or early perimenopause (less predictable menstrual bleeding but bleeding at least once every 3 months). Potential participants were excluded if they did not have an intact uterus and at least 1 ovary or were using sex steroid hormones. A total of 7 clinical sites recruited study participants: Boston, Chicago, Detroit, Pittsburgh, Los Angeles, Newark, and Oakland. The SWAN Bone Cohort included 2,365 women from 5 sites (excluding Chicago and Newark, where BMD was not measured).

### Samples.

We conducted analyses examining the relationships of average insulin resistance and rate of change in insulin resistance with the rate of change in BMD during premenopause (before FMP –2 years), the MT (FMP –1 year to FMP +2 years), or postmenopause (after FMP +2 years). Thus, we had 3 study samples, each corresponding to a midlife stage. To be included in a stage-specific sample, women needed to have a known FMP date, and 2 or more concurrent HOMA-IR and BMD measurements in that stage. Participants were censored at first use of bone-beneficial medications (hormone therapy, calcitonin, calcitriol, bisphosphonates, denosumab, and parathyroid hormone) or diabetes medications (metformin, sulfonylurea, meglitinide, thiazolidinedione, DPP-IV inhibitor, GLP-agonist, and insulin). Of the 2,365 women in the SWAN Bone Cohort, 1,151 had a known FMP date. Of these participants, 861, 571, and 693 had the requisite HOMA-IR and BMD assessments in premenopause, MT, and postmenopause, respectively. The median IQR number of visits in each midlife stage was 5 (IQR 3, 7), 2 (IQR 2, 3), and 4 (IQR 3, 6) for premenopause, MT, and postmenopause, respectively.

### Outcomes.

The outcome for analyses was midlife stage-specific (premenopause, MT, or postmenopause) annualized change in BMD (g/cm^2^ per year). At each study visit, areal BMD (g/cm^2^) at the LS and FN were measured using Hologic instruments. An anthropomorphic spine phantom was circulated to create a cross-site calibration. Boston, Detroit, and Los Angeles sites began SWAN with Hologic 4500A models and subsequently upgraded to Hologic Discovery A instruments. Davis and Pittsburgh started SWAN with Hologic 2000 models and later upgraded to Hologic 4500A machines. When a site upgraded hardware, it scanned 40 women on its old and new machines to develop cross-calibration regression equations. A standard quality control program included daily phantom measurements, local site review of all scans, central review of scans that met problem-flagging criteria, and central review of a 5% random sample of scans. Short-term in vivo measurement variability was 0.014 g/cm^2^ (1.4%) for the LS and 0.016 g/cm^2^ (2.2%) for the FN.

To quantify midlife stage-specific annualized change in BMD, we calculated the difference in absolute LS or FN BMD between the last and first available BMD measurements during premenopause, the MT, or postmenopause, and divided the difference in BMD by the number of intervening years between BMD measurements.

### Primary exposures.

The primary exposures in analyses were either average insulin resistance level or the annualized rate of change in insulin resistance over a midlife stage. Insulin resistance was assessed by HOMA-IR, quantified as fasting blood glucose (mg/dL) times fasting serum insulin (U/mL) divided by the constant 405. Insulin and glucose were both measured at 2 different central laboratories, with results calibrated for longitudinal analyses.

Insulin was measured at Medical Research Laboratory (MRL) using the Diagnostic Products Corporation assay (intra-assay coefficient of variation [CV] 8%) through the seventh follow-up visit; thereafter, it was assayed at the Clinical Ligand Assay Service Satellite (CLASS) using the ADVIA Centaur Insulin assay (intra-assay CV 1.5–2.7%). To calibrate insulin to a single lab, 400 samples from before and after the laboratory change were reanalyzed using the ADVIA Centaur assay at the University of Michigan (UM). Results from the UM were used to calibrate CLASS measurements to MRL values.

Through follow-up visit 7, glucose was measured at MRL, using a hexokinase-coupled reaction assay (Roche, intra-assay CV 1.6%); subsequent glucose measurements were performed at the UM using the ADVIA Chemistry Glucose Hexokinase assay (intra-assay CV 0.7–0.9%). A calibration equation was developed using 565 randomly selected values across the range of glucose assays. This equation was applied to covert MRL results to equivalent UM values.

Because HOMA-IR did not have a normal distribution, we base 2 log transformed it (log_2_HOMA-IR) for analysis. We then created 2 midlife stage-specific exposure variables: average insulin resistance and the annualized rate of change in insulin resistance. Average insulin resistance was calculated as the sum of all log_2_HOMA-IR measurements at study visits within the midlife stage, divided by the number of visits. Note that the arithmetic average of log-transformed HOMA-IR is mathematically equivalent to the geometric mean of raw (untransformed) HOMA-IR. Annualized change in insulin resistance was calculated by dividing the difference between the last and first log_2_HOMA-IR values within a midlife stage by the number of years between those measurements.

### Covariates.

Analyses were adjusted for age (years), race and ethnicity, BW (kg), cigarette use (yes/no), study site, and use of bone-negative medications (oral or injectable glucocorticoids, aromatase inhibitors, gonadotropin releasing hormone agonists, or anti-epileptic medications). We adjusted for bone-detrimental medication use, instead of censoring at first use (as we did with bone beneficial medications), because very few women reported taking these agents consistently over time. In contrast, bone-beneficial medications (which were used to treat osteoporosis) were used for longer intervals.

### Statistics.

Our first analysis examined the relationship of average insulin resistance over a midlife stage with the concurrent annualized rate of change in BMD. Because insulin can have anabolic effects on bone (34, 35), but bone may also become resistant to insulin’s anabolic effects in insulin-resistant states (5–7), we first visualized the functional form of the relationship between the average insulin resistance level and the rate of change in BMD using LOESS plots separately in each of the 3 midlife stages (premenopause, the MT, and postmenopause). In each stage, we found a biphasic relation with an inflection point (knot) at log_2_HOMA-IR equal to 1.5 (corresponding to HOMA-IR = 2.82). The rate of BMD change increased (or bone loss slowed) as HOMA-IR increased, up to the knot at 1.5; above that level, average log_2_HOMA-IR had no relationship with the rate of BMD change (Figure 1). To model this biphasic relationship and control for confounders, we used multivariable linear regression with a stage-specific annualized rate of change in LS or FN BMD (g/cm^2^ per year) as the dependent variable, and a 2-piece linear spline (with knot at 1.5) for stage-specific average log_2_HOMA-IR as the primary predictor. Covariates were midlife stage-specific average BW (kg), annualized change in BW (kg/year) over the midlife stage, age at the time of the first BMD/HOMA-IR measurement (years), cigarette use (yes/no) at the time of the first BMD/HOMA-IR measurement, race and ethnicity, and study site. Separate analyses were conducted in each of the 3 midlife stages.

Our second analysis examined whether the annualized rate of change in insulin resistance was associated with the rate of concurrent change in BMD. We first examined the functional form of the relationship between the 2, using LOESS separately in each midlife stage. The LOESS plot revealed a biphasic relationship in each stage (premenopause, MT, and postmenopause) with a change of slope (knot) at 0 (Figure 2). When insulin resistance was decreasing (rate of change in log_2_HOMA-IR < 0), a more positive rate of change (slower decrease) in insulin resistance correlated with a more positive rate of BMD change (slower bone loss); when insulin resistance was increasing (rate of change in log_2_HOMA-IR ≥ 0), a more positive rate of change (faster rise) in insulin resistance correlated with a more negative rate of BMD change (faster bone loss). To model this biphasic relationship and control for confounders, we used multivariable linear regression with stage-specific annualized change in LS or FN BMD (g/cm^2^ per year) as the dependent variable and a 2-piece linear spline (with knot at 0) for stage-specific annualized rate of change in log_2_HOMA-IR as the primary predictor. Covariates were as above in the first analysis.

Our final analysis examined whether the average level of insulin resistance and annualized change in insulin resistance were related to the annualized change in BMD independent of the other. For each midlife stage, we again used multivariable linear regression with stage-specific annualized change in LS or FN BMD as the dependent variable and stage-specific average log_2_HOMA-IR and annualized rate of change in log_2_HOMA-IR as predictors in the same model. Covariates were as above.

### Study approval.

Each SWAN clinical site obtained Institutional Review Board approval: University of Michigan, Ann Arbor, Michigan, USA; Massachusetts General Hospital, Boston, Massachusetts, USA; Rush University, Rush University Medical Center, Chicago, Illinois, USA; University of California, Davis, Davis, California, USA; University of California, Los Angeles, Los Angeles, California, USA; Albert Einstein College of Medicine, Bronx, New York, New York, USA; University of Medicine and Dentistry – New Jersey Medical School, Newark, New Jersey, USA; and University of Pittsburgh, Pittsburgh, Pennsylvania, USA. All participants provided written informed consent.

## Author contributions

Participant recruitment for the parent SWAN study was contributed by GAG and JAC. AS, GAG, and ASK conceived the study. AS and ASK designed the analysis. AS performed the data analysis and drafted the primary manuscript. AS, GAG, JAC, PS, and ASK critically reviewed and revised the manuscript.

## Supplementary Material

ICMJE disclosure forms

## Figures and Tables

**Table 1:**
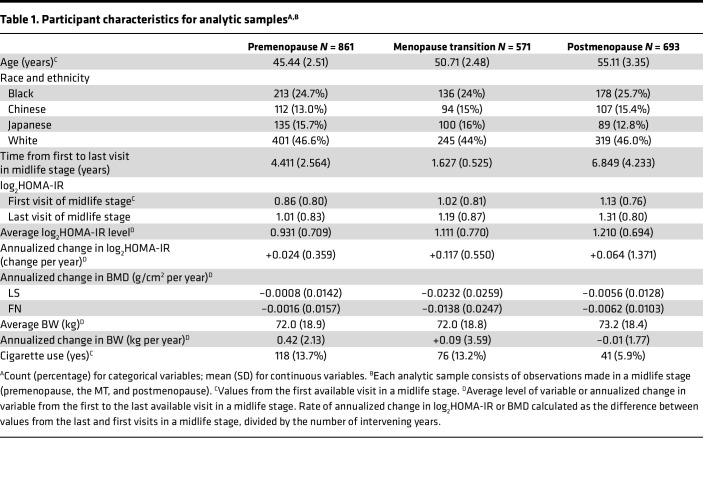
Participant characteristics for analytic samples^A,B^

**Table 2:**
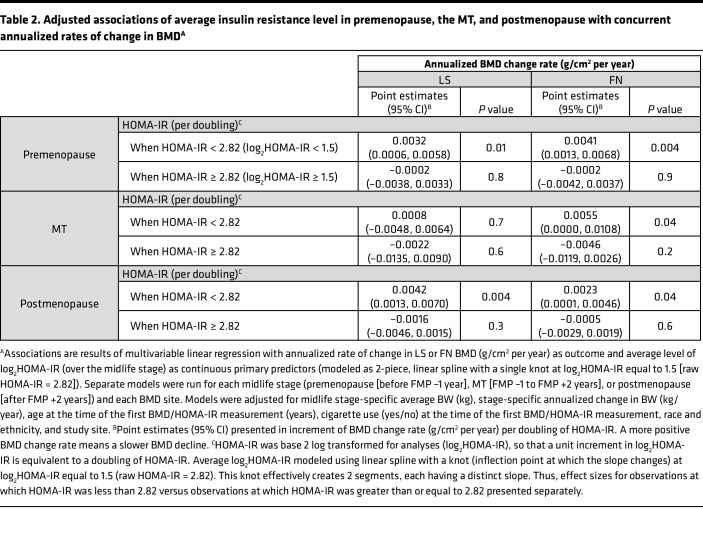
Adjusted associations of average insulin resistance level in premenopause, the MT, and postmenopause with concurrent annualized rates of change in BMD^A^

**Table 3:**
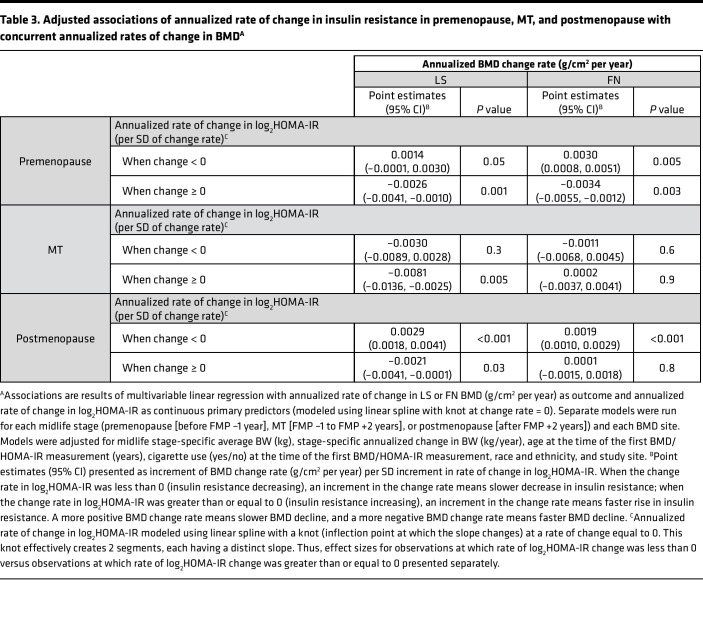
Adjusted associations of annualized rate of change in insulin resistance in premenopause, MT, and postmenopause with concurrent annualized rates of change in BMD^A^

**Table 4:**
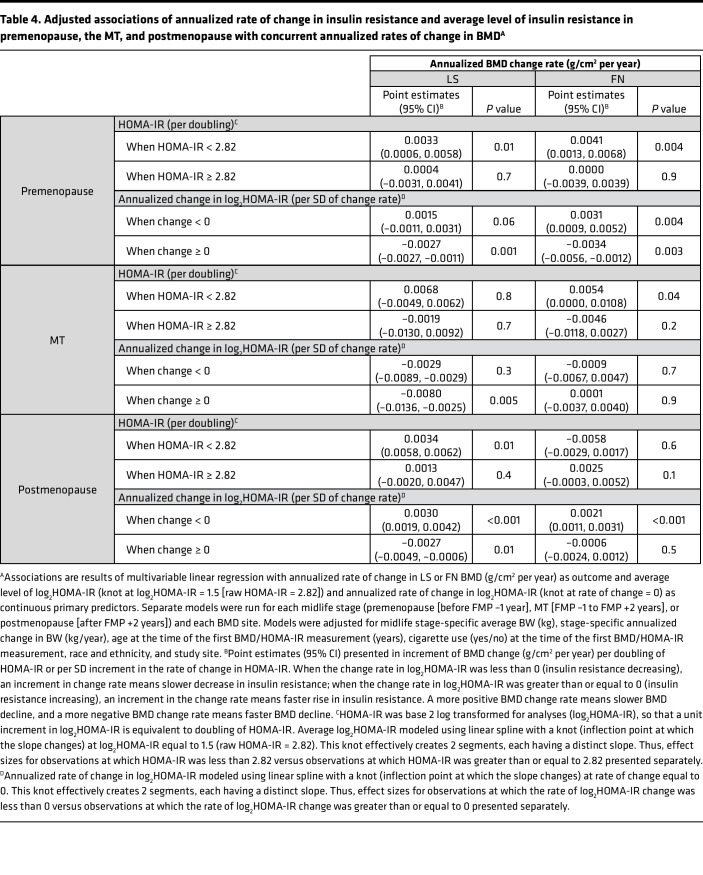
Adjusted associations of annualized rate of change in insulin resistance and average level of insulin resistance in premenopause, the MT, and postmenopause with concurrent annualized rates of change in BMD^A^

## Version 1 10/24/2022

Electronic publication
